# Argonaute 2 in Cell-Secreted Microvesicles Guides the Function of Secreted miRNAs in Recipient Cells

**DOI:** 10.1371/journal.pone.0103599

**Published:** 2014-07-29

**Authors:** Zhiyuan Lv, Yao Wei, Dong Wang, Chen-Yu Zhang, Ke Zen, Limin Li

**Affiliations:** State Key Laboratory of Pharmaceutical Biotechnology, School of Life Sciences, Nanjing University, Nanjing, Jiangsu, China; University of Torino, Italy

## Abstract

MicroRNAs (miRNAs) secreted by cells into microvesicles (MVs) form a novel class of signal molecules that mediate intercellular communication. However, several fundamental aspects of secreted miRNAs remain unknown, particularly the mechanism that governs the function or fate of exogenous miRNAs in recipient cells. In the present study, we provide evidence indicating that Argonaute 2 (Ago2) plays a role in stabilizing miRNAs and facilitating the packaging of secreted miRNAs into MVs. More importantly, Ago2 in origin cell-secreted MVs (but not in recipient cells) directs the function of secreted miRNAs. First, Ago2 overexpression clearly increased the level of miR-16 in cells transfected with a miR-16 mimic by protecting the miRNAs from degradation in lysosomes. Second, Ago2 overexpression increased the level of miR-16 in cell-secreted MVs, suggesting that Ago2 may facilitate the packaging of secreted miRNAs into MVs. Third, exogenous miR-16 delivered by MVs within the origin cells significantly reduced the Bcl2 protein level in recipient cells, and miR-16 and Bcl2 mRNA were physically associated with exogenous HA-tagged Ago2 (HA-Ago2). Finally, the effect of MV-delivered miR-16 on the production of the Bcl2 protein in recipient cells was not abolished by knocking down Ago2 in the recipient cells.

## Introduction

MicroRNAs (miRNAs) are a class of noncoding RNAs; the processed transcripts are approximately 22 nucleotides in length and regulate gene expression in plants and animals at the posttranscriptional level [1], [2]. miRNAs exert their actions through the RNA-induced silencing complex (RISC), resulting in translational repression or mRNA cleavage [3]–[5]. As an important component of RISC, Argonaute 2 (Ago2) is required for miRNA activity. Recent studies by us and others have indicated that miRNAs can be actively transported between cells through cell-secreted microvesicles (MVs) [6], [7] and that these secreted, MV-delivered miRNAs serve as a novel class of signal molecules that enter recipient cells and target their genes [7]–[9]. Accumulating evidence suggests that Ago2 is also secreted by cells into MVs and may be involved in the function of secreted miRNAs [7], [10]–[12]. In addition to forming RISC, our recent results show that Ago2 in MVs plays a critical role in protecting secreted miRNAs [11]. However, several fundamental issues regarding secreted miRNAs and their function or fate in recipient cells remain unaddressed. First, under various physiological conditions, cells secrete a variety of miRNAs or secrete miRNAs at a variety of ratios [13]–[17]. The mechanism that governs the selective secretion of miRNAs is unclear. Second, there are hundreds of miRNAs in each cell-secreted MV, and not all of these secreted miRNAs can serve as signal molecules and change the function of the recipient cells. Instead, many miRNAs are likely degraded in the recipient cells. The factors that control the fate of secreted miRNAs in recipient cells remain unknown.

In the present study, we examined the effect of Ago2 on the cellular expression level of miR-16, the packaging of miR-16 in cell-secreted MVs and the function of MV-encapsulated miR-16 in recipient cells. Our results demonstrate that Ago2 facilitates the packaging of miR-16 into MVs secreted by HeLa cells and that Ago2 in MVs keys the function of secreted miR-16 in recipient cells.

## Materials and Methods

### Reagents and antibodies

Synthetic RNA molecules, including miR-16, 5′-3Cy5.5-labeled miR-16 oligonucleotides and scrambled negative control oligonucleotides, were purchased from RiboBio (Guangzhou, China). Mouse monoclonal anti-Ago2 (ab57113) and rabbit polyclonal anti-Ago2 (ab32381) antibodies were purchased from Abcam (Hong Kong, China). Rabbit polyclonal anti-GAPDH antibody (sc-2578), mouse monoclonal anti-HA antibody (sc-7392) and Protein G Agarose (sc-2003) were purchased from Santa Cruz Biotechnology (Santa Cruz, CA). An anti-Bcl-2 (50E3) antibody was purchased from Cell Signaling (Danvers, MA, USA). Normal mouse IgG was purchased from Millipore (Cat. No. 12-371). Alexa Fluor 594-conjugated goat anti-mouse antibody was purchased from Jackson Immuno Research (Cat. No. 115-585-003). BacMam CellLight Reagents (C10507) were purchased from Life Technologies (New York, NY).

### MV isolation

MVs were isolated from cell culture medium using differential centrifugation according to previous publications [6], [7]. Briefly, cell culture medium were sequentially centrifuged at 300×*g* (30 min), 1200×*g* (30 min) and 10,000×*g* to isolate the supernatant, which was then centrifuged at 110,000×*g* for 70 min (all steps were performed at 4°C). For cell culture, 10 ug MVs were added to per 10^5^ recipient cells.

### Cell culture

Human HeLa and HEK 293T cell lines were purchased from the China Cell Culture Center (Shanghai, China). Cells were maintained at 37°C in a humidified, 5% CO_2_ incubator in Dulbecco's modified Eagle medium (DMEM) containing 10% fetal bovine serum (Gibco, Cat. No. 10099-141), 100 units/ml of penicillin and 100 µg/ml of streptomycin.

### FACS analysis of MVs

Pre-enriched MVs were incubated with exosome – Human CD63 Isolation Dynabeads (Life Technologies, Cat. No. 10606D) overnight at 4°C. Bead-bound MVs were isolated by magnetic separator. CD63 antibody (Santa Cruz, Cat. No. sc-31214) and Alexa Fluor 488 conjugated second antibody were added for incubation in 1 hour at room temperature one after another. Labeled MVs were analyzed with a FACSCalibur cytometer (Becton-Dickinson) using the FCS Express V3 software.

### RNA isolation and RT-qPCR of mRNA or mature miRNAs

Total cell RNA was extracted using TRIzol reagent (Invitrogen), and RNA from cells, MVs and immunoprecipitation products was isolated using the miRNeasy kit (QIAGEN, Cat. No. 217004). RT-qPCR was performed using TaqMan probes (Applied Biosystems) for mature miRNA or SYBR Green (Takara, Dalian, China) for pre-miRNA. Briefly, total RNA was reverse-transcribed into cDNA using AMV reverse transcriptase (Takara) and a stem-loop RT or reverse primer (Applied Biosystems). Real-time PCR was performed using an Applied Biosystems 7900 Sequence Detection System (Applied Biosystems). All reactions, including the no-template controls, were run in triplicate. After the reaction, the CT values were determined using fixed cycle threshold settings.

### Immunoprecipitation and Immunoblotting

Cells or MVs were lysed in lysis buffer (20 mM Tris-HCl, 150 mM NaCl, 0.5% Nonidet P-40, 2 mM EDTA, 0.5 mM DTT, pH 7.5) containing 1 mM NaF, 1 mM PMSF and 1% Protease Inhibitor Cocktail (Sigma-Aldrich, St. Louis, MO) for 30 min on ice. The lysates were cleared by centrifugation (16,000×*g*, 10 min, 4°C) and then immunoprecipitated with mouse monoclonal anti-Ago2 antibody or mouse normal IgG followed by Protein G-Agarose beads. After elution from the beads, RNA was prepared using the miRNeasy kit. The miRNAs associated with Ago2 were analyzed using qRT-PCR. For Western blot analysis of Ago2 or the HA tag, a rabbit polyclonal anti-Ago2 antibody or mouse monoclonal anti-HA antibody (HA probe) was used, respectively. Normalization was performed by blotting the same samples with an antibody against GAPDH.

### Immunofluorescence labeling

Cells were cultured on 8-well chamber slides. HA-Ago2 plasmid and 3Cy5.5-labeled miR-16 were transfected into HeLa cells. Lysosomes were labeled using BacMam CellLight Reagents, and 24 h later, the cells were fixed with 4% paraformaldehyde and then permeabilized with 0.1% Triton X-100 for 10 min. The cells were subsequently probed with antibodies against Ago2 followed by incubation with Alexa 350 conjugated anti-mouse antibody. To label the MVs, 293T cells treated or not treated with HeLa MVs were harvested and washed twice in PBS. The cells were then fixed, permeabilized and blocked for 30 minutes with 3% BSA. The cells were subsequently stained with mouse anti-HA antibodies for 30 min followed by detection with Alexa Fluor 594-conjugated goat anti-mouse secondary antibodies. Samples were mounted using antifade solution (Molecular Probe, Cat. No. P-7481) for observation under FV1000 laser scanning confocal microscopy (Olympus, Tokyo, Japan).

### Statistical analysis

All images of Western blots and RT-qPCR assays represent the results of at least three independent experiments. RT-qPCR was performed in triplicate. Data are presented as the means ± SD for three or more independent experiments. Differences were considered statistically significant at *P*<0.05, as analyzed using Student's *t*-tests (for paired samples).

## Results

### Ago2 protects cellular miRNA from degradation in lysosomes

**Figure 1:**
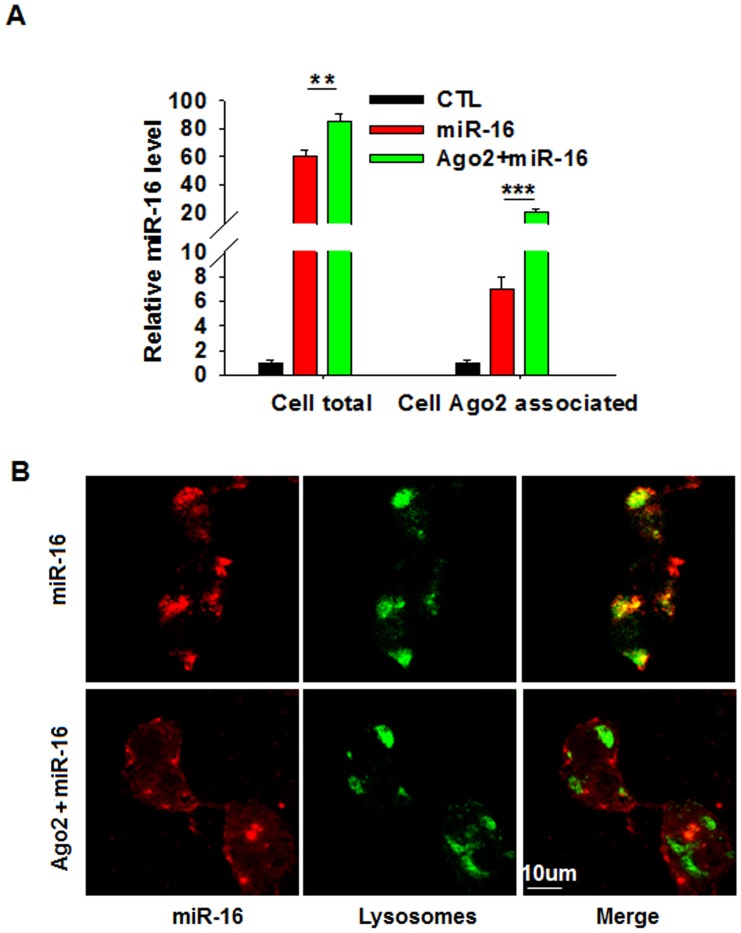
Overexpression of Ago2 can enhance co-expressed miR-16 at the cellular level. **A**, Detection of the total or Ago2-associated miR-16 level in HeLa cells co-transfected with a miR-16 mimic and Ago2-expressing plasmid. **B**, Tracing of the 3Cy5.5-labeled miR-16 in HeLa cells co-transfected or not with Ago2-expressing plasmid. Red: 3Cy5.5-labeled miR-16, Green: BacMam CellLight-labeled lysosomes. ^**^, *P*<0.01, ^***^, *P*<0.001. Scale bar = 10 µm.

To test the effect of Ago2 on miRNAs at the cellular level, we co-transfected HeLa cells with a miR-16 mimic and Ago2-expressing plasmid and assessed the cellular abundance of Ago2, miR-16 and Ago2 associated miR-16. As shown in Figure S1 and S2, Ago2 in HeLa cells was effectively overexpressed and immunoprecipitated. The total and Ago2 associated miR-16 were then determined using RT-qPCR. As shown in Figure 1A, transfection with the miR-16 mimic alone led to an increase in the levels of cellular miR-16 and Ago2-associated miR-16 in HeLa cells. However, the levels of both cellular miR-16 and Ago2-associated miR-16 were strongly increased by co-transfection with Ago2. The enrichment of cellular and Ago2-associated miR-16 by Ago2 overexpression was also supported by data tracing miR-16 in HeLa cells (Figure 1B). In this experiment, HeLa cells were transfected with the 3Cy5.5-labeled miR-16 mimic. According to the results, a majority of the fluorescent miR-16 co-localized with the lysosomes, suggesting that much of the overexpressed miR-16 does not have a biological function but is instead degraded in lysosomes. Interestingly, co-transfection with Ago2 increased the portion of fluorescent miR-16 that was not co-localized with the lysosomes. Together, these results suggest that Ago2 may increase the level of cellular miRNA during miRNA overexpression by preventing newly synthesized miRNA degradation in the lysosomes.

### Ago2 facilitates the packaging of miR-16 into cell-secreted microvesicles (MVs)

**Figure 2:**
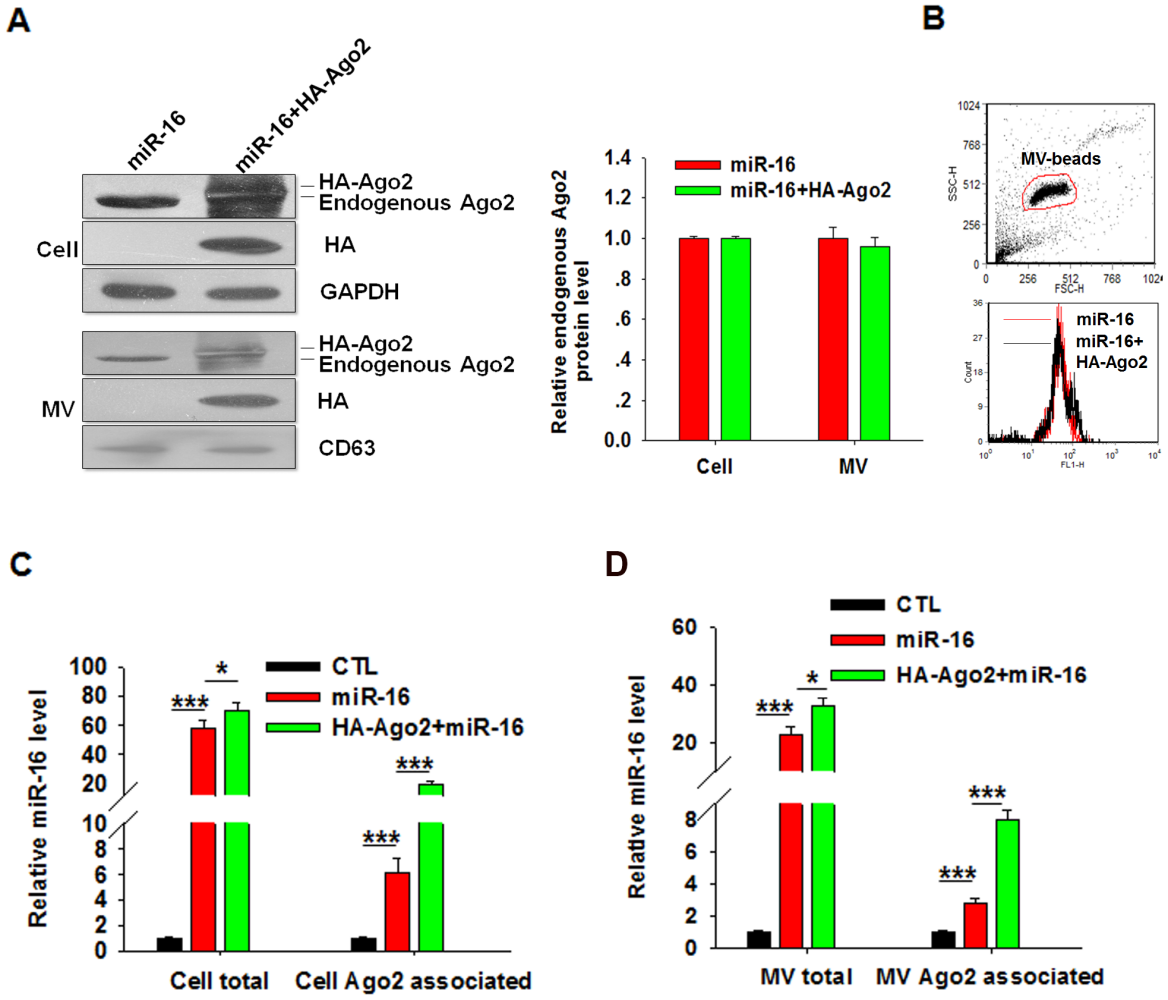
Overexpression of Ago2 can enhance the packaging of miR-16 into cell-secreted MVs. **A**, Detection of HA-Ago2 in HeLa cells and cell-secreted MVs using Western blot analysis. Cells were transfected with HA-Ago2-expressing plasmid. The right histogram represents the endogenous Ago2 protein level in miR-16 overexpressed or miR-16 and HA-Ago2 plasmid co-overexpressed cells and their secreted MVs. For cells, the level of Ago2 was normalized to GAPDH. For MVs, the level of endogenous Ago2 was normalized to CD63. **B**, FACS analysis of MVs number secreted by miR-16 transfected and miR-16 and HA-Ago2 plasmid co-transfected HeLa cells. The up scatter diagram represents beads-bounded MVs gated. The down histogram represents the analysis of CD63 expression of the gated MVs. **C**, Levels of total miR-16 and Ago2-associated miR-16 in HeLa cells. **D**, Levels of total miR-16 and Ago2-associated miR-16 in MVs. ^*^, *P*<0.05; ^***^, *P*<0.001.

We next assessed the levels of miR-16 in cell-secreted MVs following transfection with the miR-16 mimic. MVs were first isolated from culture supernatant using high-speed centrifugation, and the levels of miR-16 were assayed using RT-qPCR. In this experiment, HA-tagged Ago2 (HA-Ago2) was also overexpressed in HeLa cells. As shown in Figure 2A, strong expression of HA-Ago2 was found in HeLa cells and HeLa cell-secreted MVs, and the endogenous expression of Ago2 was not changed. In addition, as shown in Figure 2B, the overexpression of Ago2 could not change the number of secreted MVs. As expected, overexpression of HA-Ago2 significantly increased the miR-16 levels in HeLa cells (Figure 2C). These results imply that HA-Ago2 may behave similarly to normal Ago2 in terms of preventing miRNA degradation. Moreover, the overexpression of HA-Ago2 strongly increased the levels of miR-16 in MVs compared to those resulting from transfection with the miR-16 mimic alone (without HA-Ago2 expression) (Figure 2C), suggesting that HA-Ago2 may facilitate the packaging of miRNA into MVs by HeLa cells.

### Ago2 in origin cell-secreted MVs controls the function of secreted miR-16 in recipient cells

**Figure 3:**
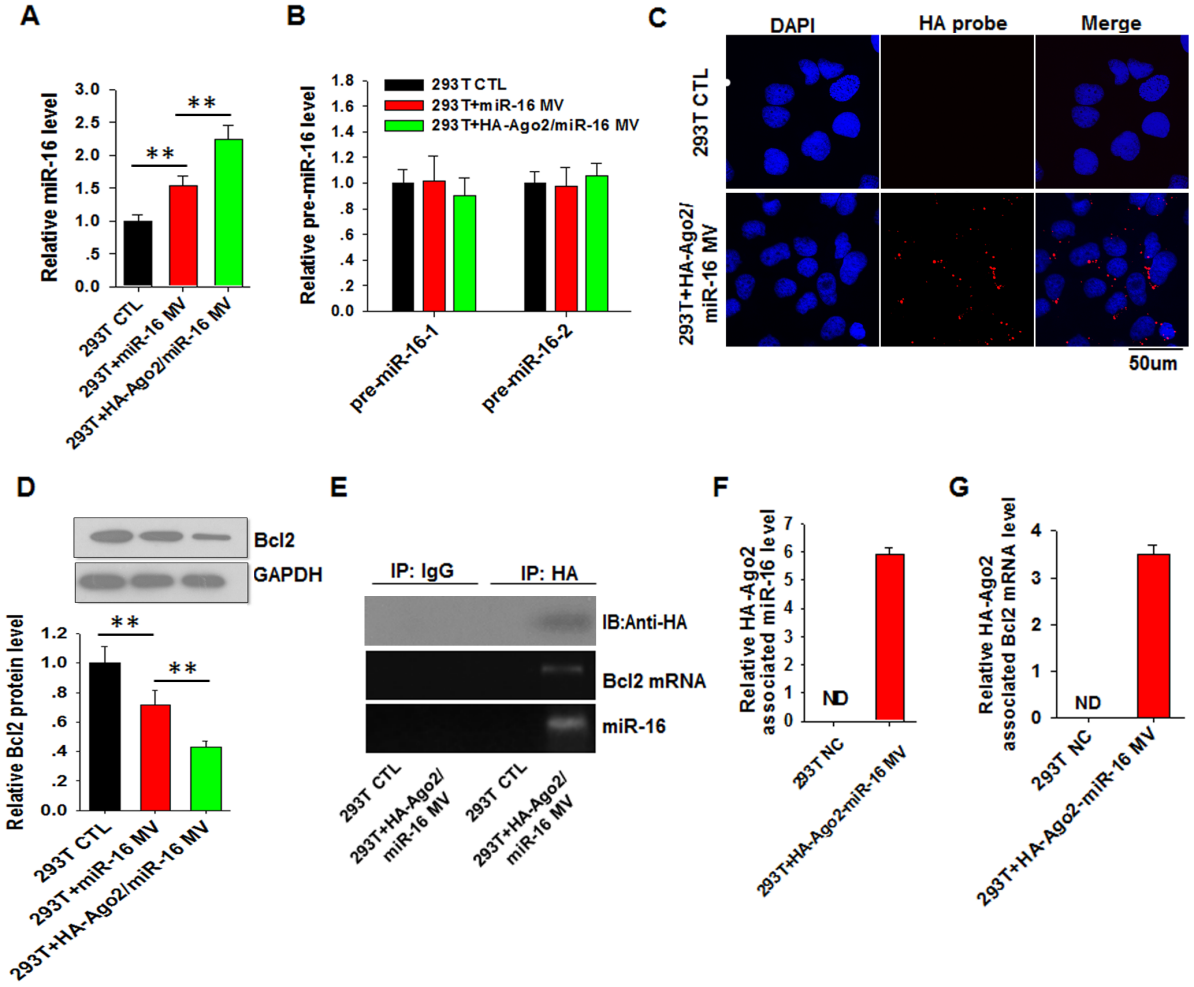
HA-Ago2 in MVs guides the function of secreted miR-16 in recipient cells. **A** and **B**, The expression levels of miR-16 (A) and pre-miR-16 (B) in 293T cells detected using RT-qPCR. 293T cells were incubated with MVs secreted from HeLa cells transfected with random oligonucleotides (CTL), miR-16 (miR-16) or co-transfected with the miR-16 mimic and HA-Ago2 plasmid (HA-Ago2/miR-16). **C**, Immunofluorescence staining of HA-Ago2 in 293T cells with anti-HA antibody. **D**, The protein levels of Bcl2 in 293T cells detected using Western blot analysis. **E–G**, The direct association of miR-16 (**E** and **F**) and Bcl2 mRNA (**E** and **G**) with HA-Ago2. HA-Ago2 was immunoprecipitated from recipient 293T cell lysate using anti-HA antibody, and the levels of miR-16 (**F**) and Bcl2 mRNA (**G**) associated with HA-Ago2 or control IgG were detected using RT-qPCR. ^**^, *P*<0.01.

To monitor the biological effect of MV-encapsulated miR-16 on a target gene in recipient cells, we incubated 293T cells with MVs isolated from HeLa cells that had been transfected with miR-16 or co-transfected with the miR-16 mimic and HA-Ago2 plasmid. As shown in Figure 3, compared with CTL, MVs isolated from HeLa cells transfected with miR-16 and from cells co-transfected with miR-16 and HA-Ago2 significantly increased the level of miR-16 in the 293T cells (Figure 3A), whereas the levels of pre-miR-16-1 and pre-miR-16-2 were unaffected (Figure 3B), indicating that the increased miR-16 observed in 293T cells is delivered by the HeLa cell MVs. In support of this conclusion, we found that HA-Ago2 was also abundant in the cytoplasm of 293T cells (Figure 3C). Western blot analysis showed that incubation with HeLa-cell MVs co-transfected with miR-16 and HA-Ago2, which contain higher levels of the miR-16/HA-Ago2 complex compared to HeLa-cell MVs transfected with miR-16, significantly reduced the protein level of Bcl2, a target gene of miR-16 [18] (Figure 3D).

To directly address whether the exogenous miR-16 was targeting the recipient-cell Bcl2, we immunoprecipitated HA-Ago2 with an antibody against HA-tag and then tested whether miR-16 and Bcl2 mRNA were associated with HA-Ago2. As shown, miR-16 (Figure 3, E and F) and the Bcl2 mRNA (Figure 3, E and G) were clearly pulled down with the HA-Ago2 complex.

**Figure 4:**
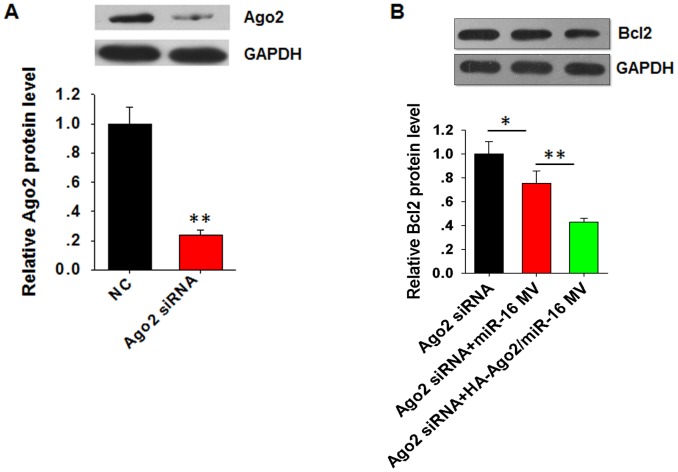
The function of secreted miR-16 in recipient cells does not require endogenous Ago2 from the recipient cells. **A**, Knockdown of Ago2 in 293T cells via Ago2 siRNA. 293T cells were incubated with MVs secreted by HeLa cells transfected with miR-16 or co-transfected with the miR-16 mimic and HA-Ago2 plasmid. **B**, Level of Bcl2 protein in 293T cells detected using Western blot analysis. ^*^, *P*<0.05; ^**^, *P*<0.01.

Given that exogenous HA-Ago2 can guide the function of its associated miRNA in recipient cells, we then asked whether the function of secreted miR-16 in recipient cells requires endogenous Ago2 from the recipient cells. In this experiment, we knocked down Ago2 in 293T cells via Ago2 siRNA and then incubated the cells with MVs derived from HeLa cells that had been co-transfected with the miR-16 mimic and HA-Ago2 plasmid. As shown in Figure 4A, the endogenous Ago2 level was reduced by more than half by the Ago2 siRNA treatment. However, the reduction of Bcl2 in 293T cells by MV-encapsulated miR-16 was not affected (Figure 4B). It is important that incubation with MVs secreted by miR-16 and HA-Ago2 co-transfected HeLa cells with higher capacity to inhibit the expression of Bcl2 in recipient 293T cells compared to incubation with MVs secreted by miR-16 transfected cells. This result supported our assumption that the function of secreted miR-16 in recipient cells did not require endogenous Ago2 from the recipient cells.

## Discussion

As a major component of RISC, Ago2 has been shown to be involved in mediating miRNA functions in various cell types [19]–[23]. Our recent study demonstrates that Ago2 plays a critical role in stabilizing miRNAs in cells and cell-secreted MVs [11]. The results of this study provide further evidence that Ago2 protects cellular miRNAs and cell-secreted miRNAs. In cells transfected with an miRNA mimic, a large portion of newly synthesized miRNA is normally transported into lysosomes and degraded. However, when an miRNA associates with Ago2, it may avoid degradation in lysosomes and therefore be available to fulfill its biological function.

Cell-released MVs serve as a major pathway for miRNA secretion [24]. As an ideal carrier for small RNA, MVs efficiently deliver secreted miRNAs into recipient cells, where these exogenous miRNAs are able to execute their function as endogenous miRNAs [25]. Our previous study showed that cells might selectively secrete miRNAs via MVs in response to various physiological or pathophysiological stimuli [7]. For example, THP-1 cells could secrete various miRNAs when challenged with LPS or free fatty acids. Previous studies by Thomson and coworkers [26] demonstrated that the majority RNAs in transfected cells were detected in lysosome, which is not accessible for loading into Argonaute as functionally miRNAs. Our results clearly suggest that Ago2 is involved in guiding miRNA sorting. In this case, the binding of miRNA to Ago2 may allow the transport of miRNAs into MVs for secretion rather than into lysosomes as non-Ago2-bound miRNAs. Although the mechanism for the selection of miRNA secretion via MVs remains unknown, the strong enhancement of miR-16 secretion via MVs in response to the overexpression of Ago2 suggests that Ago2 plays a role in facilitating the packaging of miRNAs into cell-secreted MVs.

By directly tracing the distribution of HA-tagged Ago2 in cell-secreted MVs and recipient cells, with a particular emphasis on the physical association of miR-16 and the Bcl2 mRNA with HA-Ago2, we confirmed that HA-Ago2 is biologically active and fully capable of guiding the function of MV-encapsulated miRNAs in recipient cells. In other words, miRNAs associated with Ago2 in MVs are fully functional in terms of silencing their target genes in recipient cells, and this finding was confirmed by the Ago2 knockdown experiment in recipient cells. As shown in Figure 4, the effect of miR-16 on the production of the Bcl2 protein in 293T cells, which is likely the result of exogenous miR-16 delivered by the HeLa-cell MVs, was not affected by knocking down Ago2 in 293T cells.

However, our recent study demonstrates that the differences in Ago2-bound abilities of miRNAs are significant. For example, miR-320 was observed almost not associated with Ago2, but still showed certain resistance to RNaseA. Therefore, we proposed that miR-320 in the cell-secreted MVs may be protected likely through binding with other protein(s) [11]. Moreover, studies have also shown that a significant portion of circulating miRNAs is associated with high-density lipoprotein(HDL) [27], or nucleophosmin 1 (NPM1) [13]. According to current research, Ago2, the key function protein of RISC, is predicted to prefer double-stranded pre-miRNAs. Therefore, whether the unbound Ago2 miRNAs in MVs have activity in recipient cells is dependent on whether they can load onto endogenous RISC or whether they utilize other machinery and mechanisms to recognize targeted mRNAs.

**Figure 5:**
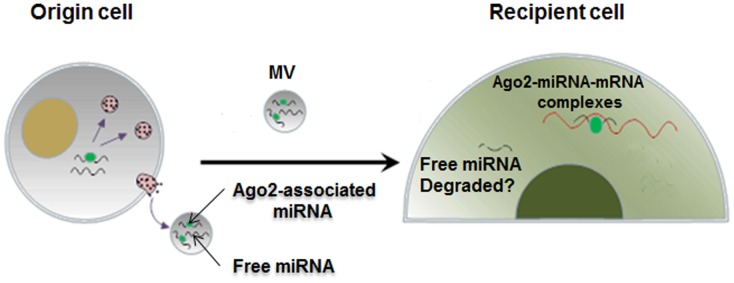
A model for the role of secreted Ago2 in guiding the function of MV-encapsulated miR-16 in recipient cells.

In summary, the present study demonstrates, for the first time, that the function of cell-secreted miRNAs in recipient cells is mediated by Ago2 associated with the miRNAs in MVs. The fate of MV-encapsulated, secreted miRNAs in recipient cells is depicted in the working model (Figure 5). First, through binding to a particular miRNA, Ago2 facilitates the packaging of this miRNA into MVs and is released by the origin cells. Second, after delivery into recipient cells, the Ago2-bound exogenous miRNA executes its function to target a recipient cell gene, whereas miRNAs that are not associated with Ago2 in the MVs are likely degraded in the recipient cells.

## Supporting Information

Figure S1 Detection of Ago2 in HeLa cells with or without Ago2 overexpression. HeLa cells were overexpressed with miR-16 (miR-16) or co-overexpressed with miR-16 and Ago2 (miR-16+Ago2). The protein levels of Ago2 were detected with rabbit polyclonal anti-Ago2 antibody.
(DOC)

Figure S2 Pull-down of Ago2 from both HeLa cells and cell-secreted MVs. HeLa cells and secreted MVs were collected and lysed. The lysates were cleared by centrifugation and then immunoprecipitated with mouse monoclonal anti-Ago2 antibody followed by Protein G-Agarose beads. After elution from the beads, Ago2 was detected with rabbit polyclonal anti-Ago2 antibody.
(DOC)
