# Supplementary material for: Argonaute 2 in Cell-Secreted Microvesicles Guides the Function of Secreted miRNAs in Recipient Cells
Source: PLoS One. 2014 Jul 29;9(7):e103599. doi: 10.1371/journal.pone.0103599 (PMC4114802; doi:10.1371/journal.pone.0103599)
Supplement: Figure S1 — Detection of Ago2 in HeLa cells with or without Ago2 overexpression. HeLa cells were overexpressed with miR-16 (miR-16) or co-overexpressed with miR-16 and Ago2 (miR-16+Ago2). The protein levels of Ago2 were detected with rabbit polyclonal anti-Ago2 antibody. (DOC) [file pone.0103599.s001.doc]

**Figure S1**


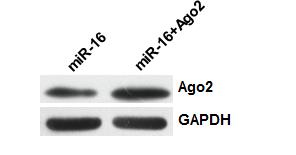


**Figure S1**. Detection of Ago2 in HeLa cells with or without Ago2 overexpression. HeLa cells were overexpressed with miR-16 (miR-16) or co-overexpressed with miR-16 and Ago2 (miR-16+Ago2). The protein levels of Ago2 were detected with rabbit polyclonal anti-Ago2 antibody.
