# Supplementary material for: Argonaute 2 in Cell-Secreted Microvesicles Guides the Function of Secreted miRNAs in Recipient Cells
Source: PLoS One. 2014 Jul 29;9(7):e103599. doi: 10.1371/journal.pone.0103599 (PMC4114802; doi:10.1371/journal.pone.0103599)
Supplement: Figure S2 — Pull-down of Ago2 from both HeLa cells and cell-secreted MVs. HeLa cells and secreted MVs were collected and lysed. The lysates were cleared by centrifugation and then immunoprecipitated with mouse monoclonal anti-Ago2 antibody followed by Protein G-Agarose beads. After elution from the beads, Ago2 was detected with rabbit polyclonal anti-Ago2 antibody. (DOC) [file pone.0103599.s002.doc]

**Figure S2**


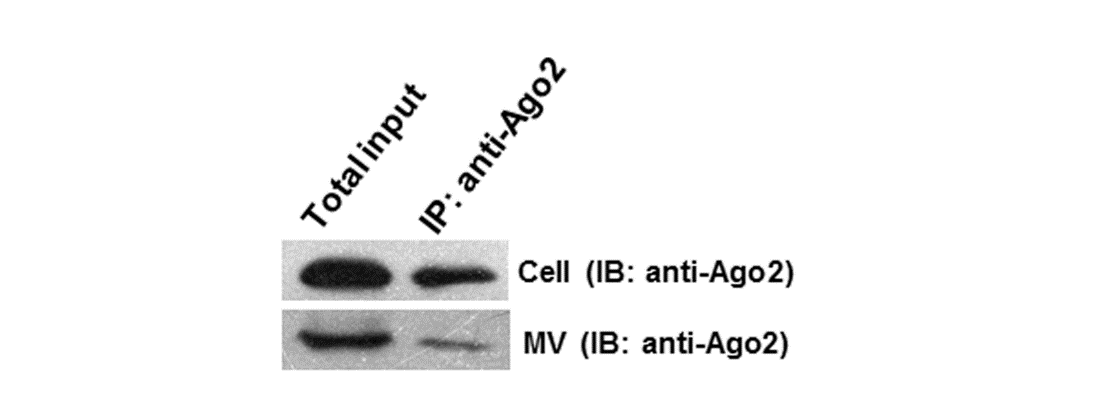


**Figure S2**. Pull-down of Ago2 from both HeLa cells and cell-secreted MVs. HeLa cells and secreted MVs were collected and lysed. The lysates were cleared by centrifugation and then immunoprecipitated with mouse monoclonal anti-Ago2 antibody followed by Protein G-Agarose beads. After elution from the beads, Ago2 was detected with rabbit polyclonal anti-Ago2 antibody.
